# Long-Range Temporal Correlations in the EEG Bursts of Human Preterm Babies

**DOI:** 10.1371/journal.pone.0031543

**Published:** 2012-02-21

**Authors:** Caroline Hartley, Luc Berthouze, Sean R. Mathieson, Geraldine B. Boylan, Janet M. Rennie, Neil Marlow, Simon F. Farmer

**Affiliations:** 1 Centre for Mathematics and Physics in the Life Sciences and Experimental Biology, University College London, London, United Kingdom; 2 University College London Institute of Child Health, London, United Kingdom; 3 Centre for Computational Neuroscience and Robotics, University of Sussex, Brighton, United Kingdom; 4 Elizabeth Garrett Anderson University College London Institute for Women's Health, London, United Kingdom; 5 Neonatal Brain Research Group, Department of Paediatrics and Child Health, University College Cork, Cork, Ireland; 6 Institute of Neurology, University College London, London, United Kingdom; Cuban Neuroscience Center, Cuba

## Abstract

The electrical activity in the very early human preterm brain, as recorded by scalp EEG, is mostly discontinuous and has bursts of high-frequency oscillatory activity nested within slow-wave depolarisations of high amplitude. The temporal organisation of the occurrence of these EEG bursts has not been previously investigated. We analysed the distribution of the EEG bursts in 11 very preterm (23–30 weeks gestational age) human babies through two estimates of the Hurst exponent. We found long-range temporal correlations (LRTCs) in the occurrence of these EEG bursts demonstrating that even in the very immature human brain, when the cerebral cortical structure is far from fully developed, there is non-trivial temporal structuring of electrical activity.

## Introduction

The EEG of very preterm babies is discontinuous, with high amplitude bursts of EEG activity interspersed within long periods of very low background activity [1]. This discontinuous pattern, known in older literature as *tracé discontinu* [2], is present between approximately 23 and 35 weeks gestational age. The pattern has a gradual age-related increase in the frequency of occurrence of the EEG bursts along with the emergence of continuous EEG oscillations throughout this age range [3]. By term gestation the EEG pattern is dominated by low frequency (1–4 Hz) oscillations with periods of nested higher frequencies [1]. In the very early preterm discontinuous EEG (23–31 weeks), bursts of nested (high-frequency) oscillations within large slow-wave depolarisations are already a prominent EEG pattern; these will therefore be the focus of this paper and will be referred to as BNOs henceforth.

Recent analyses of continuous EEG and MEG oscillations in children and adults, both at rest and during the performance of motor tasks, show power-law decay of temporal correlations in the fluctuations of oscillation amplitudes [4]–[10]. A power-law decay indicates complex temporal structure in the occurrence of events such that: (a) correlations between distant events exist and extend over longer time scales than random or short-range correlated activity (where event timing is correlated only to neighbouring previous events); and (b) the magnitude of these correlations has no distinct scale. These long-range temporal correlations (LRTCs) are characterised using estimates of the Hurst exponent [11] and it has been suggested that they reflect a complex organisational state of the brain [5]. These findings led us to the question of how early in human brain development can an EEG signature of such complex organisation be observed?

Little is known about the temporal structure of the discontinuous EEG activity in the preterm brain and what it may reveal about the nature of the processes underlying early brain development. Hitherto, the majority of quantitative studies have focused on the number of EEG bursts in a given time period and have related this measure to neonatal age and the presence of brain pathology [12]–[15]. Other studies have examined the spectral characteristics of the EEG, observing changes in spectral band power with gestational age [16]–[18] and time since birth [18], [19]. However, these studies have not examined the temporal organisation of the discontinuous preterm EEG. In this study we ask whether BNOs in neonates between 23–30 weeks gestational age are randomly distributed in time or whether their temporal occurrence possesses a complex structure characterised by LRTCs.

## Methods

### Ethics Statement

Ethical approval (NRES UK, London Central and the Clinical Research Ethics Committee of the Cork Teaching Hospitals) and written parental consent was gained for the use of the data for research purposes.

### EEG Recordings

We analysed the EEG of 11 preterm babies born between 23–30 weeks of gestation, recorded with 9 or 11 electrodes for a median duration of 21.6 hours (range 5.2–24.0 hours). EEGs were recorded at a corrected age of 23–30 weeks, at a median of one day after birth (range 0–23 days after birth, see Table 1). The EEGs were recorded on the neonatal intensive care unit at the request of the treating clinician. Four children had intracranial haemorrhages on ultrasound: all EEG recordings were classed as normal for age by an experienced clinical neurophysiologist. (For further subject details see Table 1.)

**Table 1:** Subject Information.

Subject Index	Gestational age at birth (weeks+days)	Gestational age at recording (weeks+days)	Bipolar Montage	Remaining electrodes	Recording duration (hours)	Number of events per hour (mean±s.e.m)	Ultrasound details	Further details
1	23+3	23+4	1	All	24.0	263.1±26.7	N	Normal at 6 months.
2	23+5	23+5	1	1–3,5,7,8	5.23	236.5±16.7	N	Chronic lung disease.
3	24+5	24+5	1	All	12.5	286.2±20.9	N	Normal at 10 weeks.
4	25+5	25+5	1	All	23.98	247.6±20.1	N	Normal at 9 months.
5	25+5	26+2	1	All	22.8	168.1±18.4*	A	Laser treatment for retinopathy, developed a left porencephalic cyst, chronic lung disease.
6	24+1	27+3	2	1,5–7,9,10	18.48	85.4±14.3*	B	Head CT – left transparietal shunt in situ, dilation of lateral, 3^rd^ and 4^th^ ventricles. Porencephalic cyst secondary to germinal matrix haemorrhage. Retinopathy of prematurity, chronic lung disease.
7	25+1	27+4	2	1,4,5,7,10	21.59	94.4±17.8*	C	Died from necrotizing enterocolitis.
8	26+1	28+0	2	1–8,10	22.74	92.3±7.9*	D	Chronic lung disease.
9	26+3	28+2	2	All	12.36	329.3±22.9	N	Seen at 12 weeks post term, behaving normally for age.
10	30+1	30+1	2	1,4,6–8,10	23.96	148.6±19.5	N	Discharge at term, well.
11	30+5	30+5	1	1,2,4–6,8	10.03	251.8±57.5	N	Normal at 6 weeks old.

**1** Subject information. Ages are given as weeks+days. Two different bipolar montages were used. 1 = F4-C4, C4-O2, F3-C3, C3-O1, T4-C4, C4-Cz, Cz-C3, C3-T3, 2 = F4-C4, C4-P4, P4-O2, F3-C3, C3-P3, P3-O1, T4-C4, C4-Cz, Cz-C3, C3-T3. Remaining electrodes indicates those electrodes used for analysis after artefact rejection and removal of short (<1000) IEI sequences – see methods. Electrodes are numerically indexed corresponding to the list here. Number of events per hour is averaged across all (remaining) electrodes. Subjects with haemorrhages (*) had significantly lower number of events per hour – two sample t-test, P = 1.4×10^−14^. Ultrasound details are indicated by N = normal, A = intraventricular/parenchymal haemorrhage (grade 4) left hemisphere, intraventricular haemorrhage (grade 3) right hemisphere, B = left germinal matrix haemorrhage, grade 4, C = bilateral germinal matrix haemorrhage grade 1 and cystic changes in post ventricular white matter and D = bilateral intraventricular haemorrhage involving parenchyma on right, grade 3 left, grade 4 right. Further details provide known follow up details for each subject. One child (subject 7) died of non-neurological complications of prematurity.

Babies were monitored using the NicOne digital video-EEG system (Carefusion, Wisconsin USA) with a V32, C32 or O32 amplifier. Bipolar EEG was recorded and sampled typically at a rate of 250 Hz (subjects 1–4 were recorded with the C32 or O32 amplifier with a sampling rate of 256 Hz and subject 11 with a sampling rate of 1024 Hz). Electrodes were applied to the scalp using soft paste to achieve impedances of below 5 kΩ and secured using tape and an elasticated hat. Electrodes were placed on the scalp at F3, F4, C3, C4, P3, P4, T3, T4, O1, O2 and Cz (P3, P4 were present for some older subjects only – see Table 1) according to the 10∶20 measuring system modified for neonates [20]. The EEG was recorded referred to a midline cephalic reference electrode at FCz and remontaged for bipolar derivation. The EEG was filtered: low pass filter 70 Hz, high pass 0.5 Hz and 50 Hz notch filter. Analysis was carried out on the longest artefact-free contiguous section of each time series (median section length of 18.48 hours, range 5.23–23.96 hours).

### Event Detection

**Figure 1:**
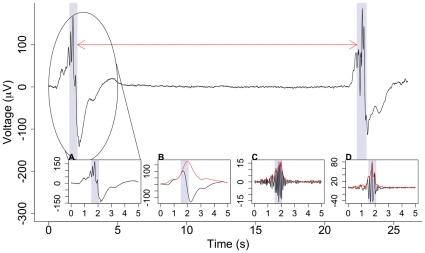
Example of nested activity detected within large depolarisations. EEG data recorded from subject 5, C4-O2. The shaded areas show detected events and the arrow indicates the inter-event interval. These events are detected from the co-occurrence of slow wave and high frequency activity. The insets show the circled signal (**A**), low-pass filtered at 2 Hz (**B**), band-pass filtered at 8–22 Hz (**C**) and with slow waves removed (**D**) along with the corresponding absolute value of the Hilbert transforms (red).

The complexity of the temporal structure of a BNO, with both a slow-wave component and a nested high-frequency oscillation, means that thresholding on the basis of amplitude alone is an insufficient detection method and a more sophisticated technique is required. The presence of a BNO was determined using a novel extraction algorithm based on the co-occurrence of a slow (0.5–2 Hz) wave and higher (8–22 Hz) frequency oscillations in each EEG channel (Figure 1). These frequency ranges were selected from the literature as they encapsulate the BNO activity [3], [21]. From the original signal x(t) three time series were obtained:

- x_1_(t), the low-pass filtered signal at 2 Hz (Figure 1b),
- x_2_(t), the band-pass filtered signal at 8–22 Hz (Figure 1c) and
- x_3_(t) = x(t)−x_1_(t), the signal with the low-frequency components removed (Figure 1d).

The third signal is required to obtain the amplitude of the high-frequency components. The Hilbert transform was applied to each of the three new signals and the absolute value taken, in order to obtain the amplitude envelopes h_i = 1,‥3_ (Figure 1b, c, d). Next, for each signal component we calculated a confidence value c_i = 1‥3_ between 0 and 1 as:
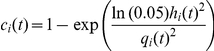
where q_1_ = 50, q_2_ = 10, q_3_ = 10 are the amplitudes of the slow and nested high-frequency components of the BNO, taken from the literature [3], [21], and yielding c_i_ values of 0.95.

Finally, we calculated:
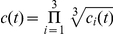
which can be thought of as a confidence value on the presence of nested activity. BNO events were defined as a contiguous section of data for which *c* was greater than or equal to the extraction threshold, taken throughout as 0.80 following sensitivity analysis. This analysis, carried out on thresholds in the range 0.65–0.95, revealed that the statistics were robust to a change in threshold provided the resulting number of events remained sufficiently large for correlations to be examined (see below). A threshold of 0.80 was chosen to correspond to high thresholds for each of the three components c_i_ (0.8>0.92^3^). The validity of this choice of threshold was then further confirmed through independent visual inspection by four different researchers. As the time series of the c values may fluctuate rapidly between values that are above and below the extraction threshold, a moving average of *c* was taken with a window size of half a second to smooth the time series. This window size was chosen as it is the largest interval that does not interfere with intervals between consecutive events – see below with reference to minimum interval size.

Consecutive events that occurred within 0.5 seconds of one another were counted as one. Similarly any events of duration less than 4/22 of a second were discounted to ensure that there was sufficient high-frequency activity (22 Hz) and at least one entire oscillation at the lowest frequency (8 Hz).

### IEI Sequences and Assessment of LRTCs

**Figure 2:**
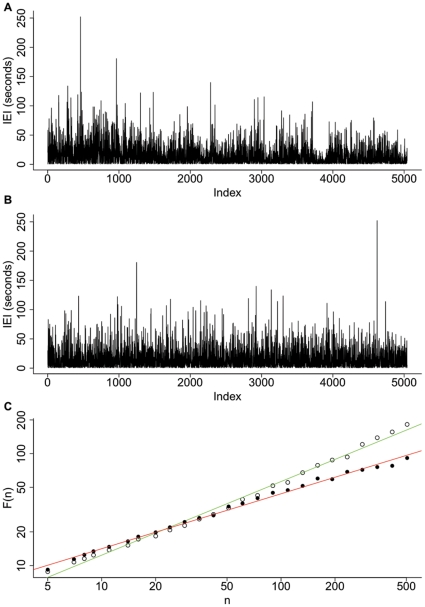
Inter-event interval (IEI) sequence and detrended fluctuation analysis (DFA) estimates. (**A**) An example of an IEI sequence – produced from the sequential ordering of IEI. This sequence is from the C3-O1 bipolar recording of subject 5. Index indicates the sequential order of the IEI. (**B**) An example of a randomly shuffled sequence for the data set shown in (**A**). (**C**) DFA plot for both sequences with window size, n, against root mean square fluctuation, F(n), open circles - DFA of the actual IEI sequence, filled circles - DFA of the shuffled sequence shown in (**B**). DFA was calculated with a maximum window size of 1/10 of the length of the sequence. For each the line of best fit is shown (green for the actual IEI sequence, red for the shuffled sequence). The Hurst exponent is estimated by the slope of the line of best fit which in these cases were H = 0.66 and H = 0.49 for the IEI and shuffled sequences respectively.

BNO events were detected in all EEG channels in all 11 preterm subjects. From the detected events we calculated the period between events - the inter-event intervals (IEI, see Figure 2). These were taken in order of occurrence in the EEG time series to obtain IEI sequences. The presence of LRTCs in sequences of IEI was assessed through estimation of the Hurst exponent, H. A process with no or short-range temporal correlations (for example white noise) has H∼0.5, whereas an exponent of 0.5<H<1 indicates persistent long-range correlations in the data. The Hurst exponent characterises self-similarity in the signal, which is also captured in the autocorrelation function of the signal. A process is said to have long-range correlations (power law decay of temporal correlations) if the autocorrelation function 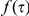 is of the form 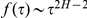 as 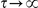 where 0<H<1 is the Hurst exponent [22] and τ is the time-lag. The Hurst exponent is also therefore, by the Wiener-Khinchin theorem, equivalent to the exponent of the power spectral density of the signal [22]. There are a number of methods for estimating the Hurst exponent and, since each method provides a biased estimate (see for example [11]), it is recommended practice to check consistency of results using two methods [22]. We used detrended fluctuation analysis (DFA) [23] and the Whittle estimator [24] to estimate the Hurst exponent of the IEI sequences. These methods have been found to produce the most accurate estimates of the Hurst exponent [11]. Further, DFA provides an estimate through analysis in the temporal domain while the Whittle estimator is a non-graphical method operating in the frequency domain. Sequences of length less than 1000 IEI were excluded from analysis to ensure robust estimates of the long-range correlations (see Table 1). As the total recording length and the number of detected BNOs differed between subjects, we also calculated the exponents for the sequences of the first 1000 IEIs (the minimum sequence length required for analysis). This allowed direct comparison of subjects by controlling for the number of BNOs.

### DFA Analysis

DFA analysis of IEI was carried out as has been described previously [23], [25]. Briefly, the signal (i.e. the sequence of IEIs) is first integrated and then divided into boxes of equal length, n. For each box, a least-squares fit to the data is found and the integrated signal is detrended by subtracting this local trend in each box. The root mean square fluctuation of this detrended signal, F(n), is given by:
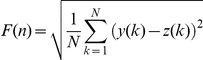
where y is the integrated time series, z is the local trend and N is the length of the signal. This process is repeated for different window sizes and the average fluctuation is compared to box size on a double logarithmic plot. A linear relationship indicates the presence of scaling of the detrended fluctuations over all box sizes and the slope of the line of best fit is the DFA exponent (see Figure 2c).

The minimum window size was set to 5 IEI, following sensitivity analysis. The maximum window size was set to one tenth of the length of the IEI sequence (the recommended maximum window size [26]), with 25 different window sizes equidistantly placed on a logarithmic scale. 25 ensures that all window sizes are distinct even for the smallest length sequences. To look at the presence of scale invariance over a broader range of time scales we also carried out DFA with a maximum window size of a quarter of the length of the IEI sequence. These results were compared with those obtained using a maximum window size of one tenth. Analysis was carried out using the Matlab code of McSharry [27].

### Whittle Analysis

To obtain robust results the Hurst exponent for each IEI sequence was also estimated using Whittle analysis. The Whittle estimate was calculated [11] as the value of η which minimizes the function Q, where Q is given by:
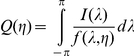
where λ is a frequency, 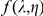 is the spectral density at frequency λ, 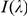 is the periodogram given by 
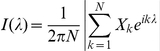
 and N is the number of terms in the time series X. For calculations we used the function FDWhittle in the R package fractal with sdf.method = “wosa”−Welch's overlapped segment averaging.

### Statistical Analysis

The Hurst exponent of each IEI sequence was compared with the Hurst exponents of 5,000 new sequences generated by randomly shuffling the original sequence, a process that destroys any LRTCs present. For each subject, and for both estimates of the Hurst exponent, the distribution of IEI exponents was compared to the distribution formed from combining all shuffled exponents for that subject, using the nonparametric one sample Wilcoxon signed rank test.

## Results

We assessed the presence of BNOs in 11 preterm subjects (23–30 weeks gestational age at birth; 9/11 EEG records obtained from subjects aged 23–28 weeks). The average number of detected events per hour for each subject is given in Table 1 and ranged from 85.4 to 329.3. The number of events did not vary with corrected age (gestational age plus time since birth), but subjects with cerebral haemorrhages (see Table 1) had significantly lower event rate (110.1±38.9 events per hour), compared to subjects without brain haemorrhage (251.7±55.1 events per hour), Wilcoxon signed rank test (P = 2.46×10^−10^).

**Figure 3:**
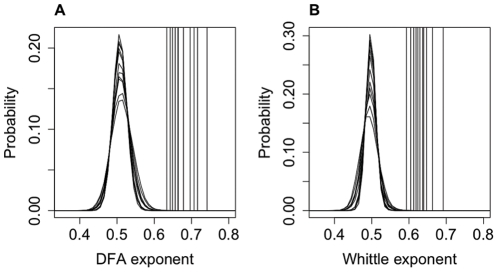
Comparison of the DFA and Whittle exponents with the shuffled data. The DFA (**A**) and Whittle (**B**) exponents for each subject (vertical lines, averaged across channels) are clearly distinct from the pooled probability distributions of exponents formed from 5000 shuffled sequences. The exponents from the IEI sequences were found to be significantly different from the shuffled distributions (P<0.001) using the one sample Wilcoxon signed rank test, indicating LRTCs in the IEI sequences of all subjects studied. The data is plotted here using the same format as Figure 4 [10].

The presence of LRTCs in the occurrence of BNO events was assessed through two estimations of the Hurst exponent of the IEI sequences. Figure 2 shows a typical IEI sequence and the corresponding resultant DFA plot, which in this case gave an exponent of H = 0.66. A total of 80 valid IEI sequences, out of a possible 98 sequences, were analysed from across the 11 subjects. The other 18 IEI sequences were rejected either due to artefacts or, in most cases, as the length of the IEI sequences was less than 1000 – see Table 1. The average Hurst exponent of all IEI sequences analysed was 0.68 (DFA with a maximum window size of 1/10 of the length of the data, range 0.55–0.81) and 0.63 (Whittle, range 0.53–0.77). There was no difference in Hurst exponents between subjects with and without cerebral haemorrhage in either DFA (P = 0.07, Wilcoxon signed rank test) or the Whittle estimate (P = 0.06, Wilcoxon signed rank test). The exponent of each IEI sequence (by subject and electrode) was compared with the exponents of 5,000 new sequences generated by randomly shuffling the original IEI sequence. An example of a shuffled sequence compared with an actual IEI sequence is shown in Figure 2 along with the corresponding DFA plots. The shuffled exponents had distributions with mean±standard deviation of 0.510±0.023 (DFA) and 0.496±0.018 (Whittle), consistent with the theoretical asymptotic value of H = 0.5 for uncorrelated noise. Figure 3 shows the pooled shuffled distributions and the average exponent values for each subject. The exponents for the actual IEI sequences are clearly distinct from the shuffled distributions. For each subject, and for both estimates of the Hurst exponent, IEI exponents were found to be significantly different from those of shuffled distributions (P<0.001), indicating the presence of LRTCs in the temporal distribution of events of every subject studied.

**Figure 4:**
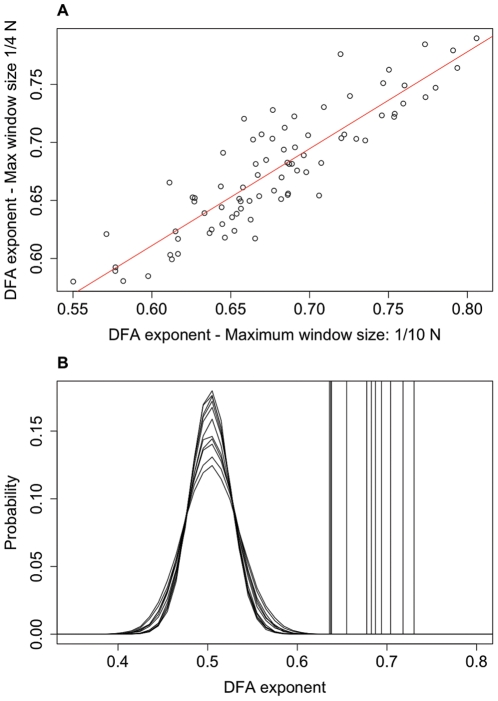
Extension of DFA to longer window sizes. Extending DFA from a maximum window size of 1/10 of the length of the signal (N = the length of the signal) to a maximum window size of 1/4 of the length of the signal showed a strong correlation between exponents (**A**) with R^2^ = 0.79 indicating that the LRTCs apply to this broader range of time scales. DFA exponents (**B**), calculated with a maximum window size of 1/4, for each subject (vertical lines, averaged across channels), are again clearly distinct from the pooled probability distributions for the shuffled sequences.

It is recommended practice to only apply DFA up to a maximum window size of 1/10 of the length of the signal [26]. It is possible to extend the range of timescales considered by increasing the maximum window size but in doing so the statistics of the fluctuations for large window sizes become less robust and so the results should be taken with caution. However, if there is a strong correlation between the exponent calculated with larger window sizes and that calculated for the more conservative estimate with window sizes up to 1/10 of the data length then this provides evidence that the LRTCs observed apply to a broader range of time scales. We carried out a further DFA analysis up to a maximum window size of 1/4 of the length of the IEI sequences. We found a strong correlation, with R^2^ = 0.79, between the DFA values calculated with a maximum window size of 1/10 and those found with a maximum window size of 1/4 (see Figure 4A). Comparison with shuffled distributions showed that the exponents calculated with a maximum window size of 1/4 are distinct from the shuffled distributions (Figure 4B) and IEI exponents were found to be significantly different from the shuffled distributions (P<0.001).

**Figure 5:**
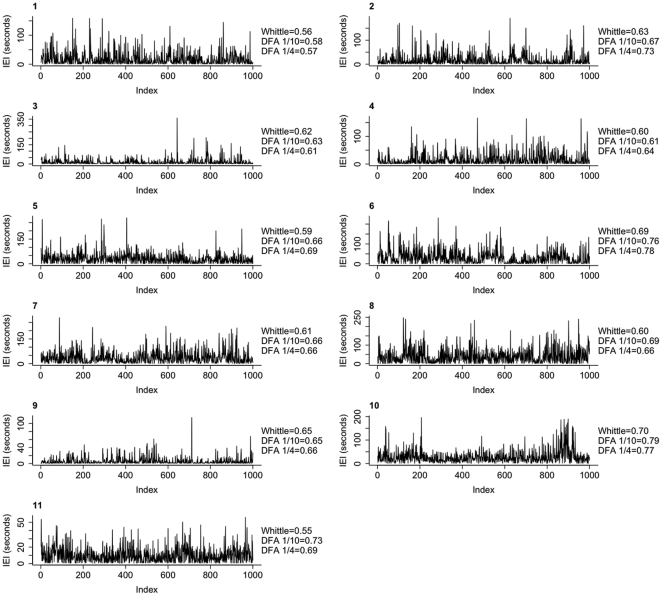
Examples of inter-event interval (IEI) sequences from each of the 11 subjects. Each sequence consists of the first 1000 IEIs from the recording at F4-C4 for each subject (1–11). The corresponding Whittle and DFA exponents for these fixed length sequences are indicated. DFA 1/10 is the exponent calculated with a maximum window size of 1/10 of the length of the data (which in this case is always 100 IEIs). DFA 1/4 is the exponent calculated with a maximum window size of 1/4 of the length of the data (which in this case is always 250 IEIs).

**Figure 6:**
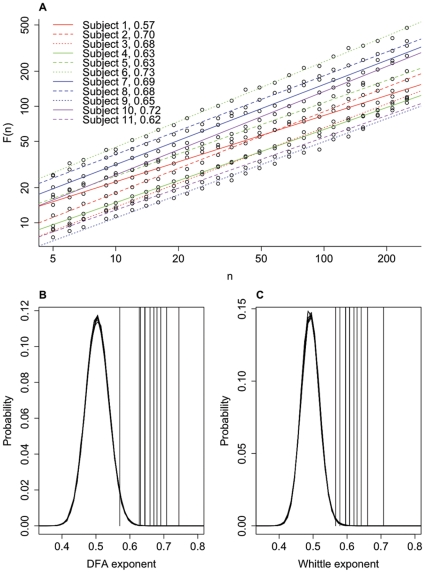
Averaged DFA plots and comparison with shuffled distributions for fixed length IEI sequences. (**A**) Average (across channels) DFA plots of window size, n, against root mean square fluctuation, F(n), for each subject for the first 1000 IEIs. DFA is calculated up to a maximum box size of 1/4 of the length of the signal i.e. 250. Exponents are as indicated. (**B,C**) Averaged DFA (**B**) and Whittle (**C**) exponents (vertical lines) for each subject from sequences of the first 1000 IEIs, along with the probability distributions of the shuffled data.

**Figure 7:**
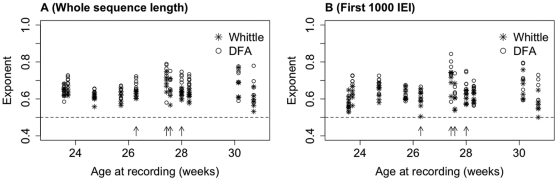
Hurst exponent estimates in relation to subject age. Whittle (stars) and DFA exponents (circles) were calculated for each subject for the whole IEI sequence (**A**) and fixed length sequences of the first 1000 IEIs (**B**). Each point is the exponent calculated for a single IEI sequence from a single channel. These are plotted against the corrected age (gestational plus time since birth). Arrows indicate subjects with intracranial haemorrhages (Table 1). DFA exponents are calculated using a maximum window size of 1/4.

As signal lengths and the number of BNOs within the signal differed between subjects, we analysed sequences of 1000 IEIs in order to better compare the exponents between subjects. For each subject and channel, both estimates of the Hurst exponent were calculated for the first 1000 IEI recorded at that channel. Figure 5 shows examples of such IEI sequences, along with the corresponding DFA and Whittle exponents. Due to the strong correlation observed above between DFA exponents calculated with different maximum window sizes, DFA was calculated with a maximum window size of 1/4 of the length of the signal. Figure 6 shows the DFA plots for each subject, averaged across all channels, along with comparison of actual exponents and the probability distributions of shuffled sequences for these short fixed length sequences of 1000 IEI. For these fixed length IEI sequences the mean estimates of the Hurst exponent were 0.66 (DFA with a maximum window size of 250, range 0.53–0.84) and 0.62 (Whittle, range 0.50–0.76). Comparison of these exponents with the pooled exponents for the shuffled distributions showed them to be significantly different (P<0.001). Figure 7 shows the exponents for the whole IEI sequences (A) and the fixed length first 1000 IEI sequences (B) plotted with respect to corrected age at the time of the recording.

## Discussion

These findings provide, to our knowledge, the first demonstration of LRTCs in the temporal sequence of bursts of high-frequency oscillations nested within large slow-wave depolarisations, in the preterm human brain. We have focussed on the discontinuous EEG and the BNOs that dominate the EEG activity in the age group studied (less than 31 weeks gestational age), and although this study analysed a small number of subjects, LRTCs in the IEIs were consistently observed in all subjects. The subjects in this study were born at 30 weeks of gestation or less, and four of the subjects had significant intracranial complications of prematurity detected on transcranial ultrasound. It should further be noted that even without the overt brain injuries that can be identified by neonatal ultrasound abnormalities, important clinical outcomes, such as low IQ [28] and special educational needs [29], are much more prevalent in the extreme preterm population [30]. Thus we cannot assume that the EEG dynamics we have detected will be present in a healthy (foetal) brain at this age. Recent new techniques using MEG have enabled the recording of foetal brain activity in utero [31] and such recordings may enable exploration of the temporal dynamics of the foetal brain in the future. Nonetheless, examination of the EEG in this preterm population has provided a unique insight into the dynamics of the very immature human brain, which we show displays complex organisation even at these very low gestations.

Our data extraction method was based on predefined amplitude and frequency criteria (taken from the literature [3], [21]) for detecting high-frequency oscillations nested within slow EEG activity. Due to this constraint we did not analyse event amplitude or duration, but rather the data was analysed as a temporal series of events from which the temporal structure of the IEI sequence was ascertained [25]. During the recording period the subjects were connected to a ventilator and subject to normal nursing and medical care. Trends in data sets that result from extraneous factors can lead to erroneous estimation of the Hurst exponent [26]. To look for such effects we examined the behaviour of the detrended fluctuations looking for loss or disruption of the linear trend across window sizes. Underlying trends in the data cause ‘crossover’ points [26] between groups of window sizes in the DFA where the detrended fluctuations follow different trends. For examples of such data see [26]. The data presented here was linear throughout and did not show crossover points; i.e. the same scaling was observed across all window sizes (see Figures 2C and 6A for examples), suggesting the Hurst exponent estimate was not affected by external stimuli.

While some studies have analysed the spectral characteristic of the preterm EEG and how this varies with age [16]–[19], clinical studies of the distribution of events in discontinuous EEG patterns in the premature population to date have only examined maximum or mean inter-burst intervals (defined as periods of silence in *all* electrodes) [12]–[15]. Therefore the presence of LRTCs observed here reveals a significantly greater complexity in the preterm EEG than previously appreciated. Studies have shown that neurological prognosis is worse in babies with less frequent EEG bursts [12]. Consistent with this, we observed that the 4 subjects with intracranial haemorrhages had a significantly lower number of BNO events per hour. However, we did not observe a difference in Hurst exponents between subjects with and without haemorrhages, indicating that despite the lower event frequency the temporal complexity of event occurrence is maintained. Further investigations will be required to establish whether the Hurst exponent is affected by pathologies in the very premature brain and whether, if differences are observed, these translate into later functional impairments.

In this data set there is no clear effect of age at recording on the Hurst exponent (see Figure 7) but it should be noted that the time since birth has not been controlled for; a larger study with correction for time of recording since birth will be required to make more definitive statements regarding the relationship between the Hurst exponent, gestational age and time since birth. This is important because recent longitudinal studies have reported changes in preterm neonatal EEG patterns with respect to *time since birth*, including changes in band power [18], [19] and decreases in the duration of inter-burst intervals [19]. These studies analysed either continuous recordings over the first few days of life [18] or recordings from the same subject for a short period on each consecutive day during the first few days of life [19]. The data we analysed here were single recordings for each subject at a time after birth dependent on clinical decisions. In order to study the effects of extra-uterine aging on the Hurst exponent further studies are required with longitudinal data sets.

The EEG recordings studied here were AC-coupled and used the conventional high-pass filter of 0.5 Hz. Recent DC-coupled EEG of preterm subjects has revealed very slow (0.1–0.5 Hz) spontaneous activity patterns with nested oscillations at many higher frequencies, known as spontaneous activity transients (SATs) [32]–[34]. SATs are thought to correspond to the delta-frequency activity (with nested higher frequencies) observed in the conventional EEG recordings [32]. Thus, in characterising the temporal distribution of BNOs it is likely we are examining the distribution of high-frequency oscillations nested within SAT events. Future analysis of DC-coupled recordings would be required to confirm this directly. The nested oscillations observed in conventional recordings, and by extension SAT events, have been suggested to be equivalent to spontaneous activity observed in the early developing rat brain [33]. Khazipov et al., [35] noted the similarity between spindle bursts evoked in the rat pup (postnatal days 1–8) somatosensory cortex and nested oscillatory activity in the human preterm brain (reviewed in [36], [37]). Spindle bursts are often associated with spontaneous movement and myoclonic twitches. This pattern of activity persists, although is reduced, following spinal cord transection and is thus not entirely dependent on movement-triggered sensory feedback, but can also occur as the result of spontaneous intrinsic brain events [35]. Experimental results in rats suggest that the subplate (a transient population of neurons that constitutes a prominent anatomical structure [38] and provides active input to the cortex during the period of development studied here [39]) plays a fundamental role in the generation of nested oscillatory activity through amplifying thalamic input to the developing cerebral cortex [40], [41]. Disruption of subplate activity causes long-term alterations in cortical connectivity [39], [42]. Additionally, early spontaneous network activity is thought to play a vital role in normal connectivity development [43] and the development of functional synaptic pathways [37]. Future studies will explore in humans the relationship between the cortical subplate, spontaneous neuronal activity and the temporal distribution of BNOs. Furthermore, future studies should investigate the temporal structure of spontaneous activity in animal models and explore whether disruption of any temporal structure leads to a functional alteration in the formation of cortical connections.

The values of the Hurst exponents recovered from our data are comparable with those observed using a similar approach of inter-spike interval sequence analysis of single unit data recorded from human hippocampus during epilepsy surgery [44]. They are also in the range of those values described for amplitude fluctuations in MEG and EEG oscillations of adults and children [4]–[10]. Significantly, however, whereas the temporal organisation of fluctuations of amplitudes may relate to a form of avalanche dynamics [45] (in which cascades of network activity – termed neuronal avalanches - recorded in local field potentials in vivo and in vitro are characterised by a power law distribution of size [46]–[49]) our findings suggest an alternative, but no less interesting, form of complex temporal dynamics in which discrete events exhibit a power law in their distribution of IEIs. This pattern may be indicative of some form of relaxation dynamics such as that observed in far-from-equilibrium systems near a phase transition [50]. Empirical evidence in support of the presence of long-range correlations in burst occurrence in neuronal systems comes from Segev et al. [51], who analysed the inter-burst intervals of spontaneous synchronous bursting activity of in vitro neural activity that is similar, albeit without the more complex structure of nested oscillations, to the activity observed here. The complex spatial dynamics of nested oscillations (theta-beta/gamma), which appear in the second postnatal week in rats (a period when the cortex is still under development) and which organise as neuronal avalanches with long-range *spatial* correlations have been suggested to provide a template important for cortical maturation and neuronal circuit formation [47]. LRTCs observed here in the temporal occurrence of BNOs might also play an important role in brain formation. Demonstrating the presence of LRTCs in very early brain EEG activity is a first crucial step in understanding the dynamical structure of preterm neuronal activity in humans, and will stimulate further research into how nested oscillatory activity shapes brain development.
